# Spatially explicit poisoning risk affects survival rates of an obligate scavenger

**DOI:** 10.1038/s41598-018-22632-y

**Published:** 2018-03-12

**Authors:** A. Monadjem, A. Kane, A. Botha, C. Kelly, C. Murn

**Affiliations:** 1 All Out Africa Research Unit, Department of Biological Sciences, University of Swaziland, Private Bag 4, Kwaluseni, Swaziland; 2 Department of Zoology & Entomology, Mammal Research Institute, University of Pretoria, Private Bag 20, Hatfield, 0028 Pretoria, South Africa; 3 School of Biological, Earth and Environmental Sciences, Cooperage Building, North Mall Campus, University College Cork, Cork, Ireland; 4 Endangered Wildlife Trust, Modderfontein, South Africa; 5 Wildlife ACT Fund Trust, Gardens, Cape Town, South Africa; 6 Hawk Conservancy Trust, Andover, Hampshire, SP11 8DY England; 7 School of Biological Sciences, University of Reading, Berkshire, RG6 6AS England

## Abstract

Obligate scavengers such as vultures provide critical ecosystem services and their populations have undergone severe declines in Asia and Africa. Intentional poisoning is a major threat to vultures in Africa, yet the impact on vulture populations of where poisoned carcasses are positioned is not known. We used re-sightings of 183 African white-backed vultures captured and tagged in two regions of South Africa, some 200 km apart, to estimate spatial differences in relative survival rates across life stages. Juvenile survival rates were similar in the two regions, whilst subadult and adult survival rates differed significantly. Using agent-based modelling, we show that this pattern of relative survival rates is consistent between regions that differ in intensity of poisoning, despite the proximity of the two regions. This may have important consequences for vulture conservation and the targeting of conservation efforts, particularly with regard to the efficacy of “vulture safe zones” around vulture breeding populations.

## Introduction

As the only obligate avian scavengers, vultures are important for ecosystem health and functioning^1–3^, yet their populations have been severely depleted by a variety of factors that include poisoning (toxicosis), electrocution by power infrastructure, and large-scale modifications to the landscape^1,4–6^. Amongst these, toxicosis, both intentional (illegal) and non-intentional (usually not illegal), is thought to be the single most important contributor to population declines of vultures^1,7^. For example, the widespread occurrence of carcasses containing veterinary drugs was the key mortality factor responsible for the collapse of south Asian vulture populations where, due to unintentional poisoning by the veterinary drug diclofenac, millions of birds died^8,9^. Furthermore, the effect of increased mortality on population dynamics is typically sensitive to the affected age class in long-lived species^10^; in vultures and storks, it is adult mortality that most affects future population trends^11,12^. The problem, however, is far more pervasive and may involve not just nonsteroidal anti-inflammatory drugs (such as diclofenac) but a whole host of veterinary pharmaceuticals including antibiotics^13^.

Intentional (and illegal) poisoning is also a major contributor of vulture mortality across the globe. For example, in Spain, which is home to 90% of European vultures, over 4,000 vultures of four species were illegally poisoned in the two decades between 1990 and 2010^14^. Similarly, African vulture populations are experiencing rapid declines and most species are now at risk of extinction^15^, with poison use being a major contributor to vulture deaths^7,16,17^. Indeed, because *Gyps* vultures are social foragers and often respond in large numbers to feeding opportunities discovered by other vultures or scavenging raptors^18^, hundreds of birds can be killed by a single, poisoned carcass^16^. Counts of over 400 dead vultures have been made at poisoned elephant (*Loxodonta africana*) carcasses in south-central Africa, with eleven reported incidents of sentinel poisoning in the region over a two-year period^16^. Sentinel poisoning occurs when carcasses are intentionally poisoned to kill vultures in an attempt to mask poaching activities from field rangers and law enforcement officers. Vultures also appear as fetish in traditional markets across the continent, with six vulture species ranking in the top 19 conservation priority bird species being traded in Africa^19^. Although the exact methods used to trap vultures for such traditional markets are not known, at least some of them would have been poisoned^20^.

Despite the large number of poisoned vultures that have been reported, the impact of these mortalities on the population dynamics of vultures has not received much attention. However, a recent study modelled the persistence of a population of 2400 African white-backed vultures (*Gyps africanus*; AWbV) in response to a range of scenarios that varied in rates of poison-induced mortality^21^. This study reported that in six of seven scenarios the population declined and in two cases the population was extirpated in 50–60 years^21^, highlighting that vulture populations are extremely susceptible to poisoning-induced mortality. However, AWbVs range widely^22^ and the risk of poisoning-induced mortality is spatially variable^7^. As a result, the impact of poisoning on the demography and persistence of AWbV populations in different areas is unknown but such knowledge is essential to understanding the impacts of continued poisoning that varies spatiotemporally and in intensity, and how targeted conservation actions can be most effective to minimize these impacts.

Green *et al*. developed a demographic model to show that even a small proportion of carcasses contaminated with nonsteroidal anti-inflammatory drugs was sufficient to explain the catastrophic decline of vultures on the Indian sub-continent^8^. However, this model was not spatially explicit, in that the authors did not include “location” (of poisoned carcasses) as a feature in the model but rather assumed that the distribution of these carcasses would be spatially homogenous. Understanding spatially variable impact of poisoning is important because areas that are vital for vulture conservation (such as breeding colonies, for example) might be at a higher risk; identifying these spatially explicit risks is thus a priority.

Our specific objectives were to: (i) quantify age-specific survival rates of AWbV in two regions of South Africa; (ii) construct population trajectories of AWbV for these two regions; and (iii) provide a plausible explanation for any observed differences. To address these objectives, we start out by showing differences in survival rates of AWbV across the two regions and across different age groups. We then construct a set of population dynamics models to predict long-term population trends over time and test their sensitivity to poisoning at different frequencies. Finally, we construct an agent-based model to test whether differential poisoning rates can effectively explain these differences in survival rates.

## Results

### Re-sightings

**Figure 1:**
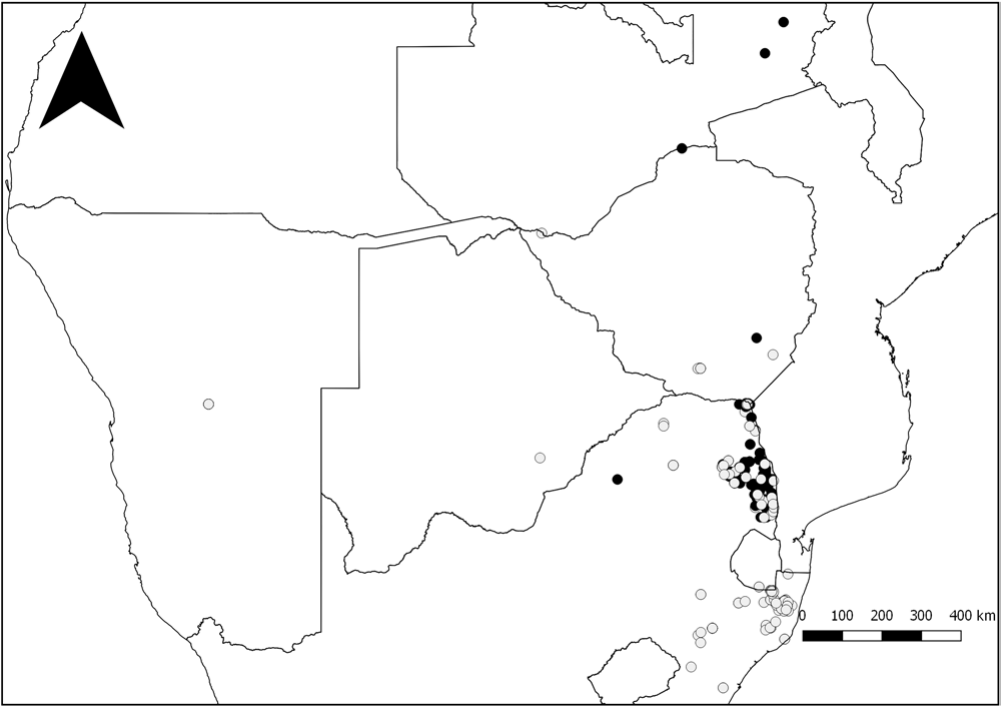
Map showing the locations of all re-sightings of African white-backed vultures tagged in this study. The black and grey circles represent individuals that were originally tagged in the Greater Kruger National Park and KwaZulu-Natal regions, respectively. The number of birds tagged in each region in each year is presented to Table S1. This map was created in Quantum GIS (Quantum GIS Development Team (2016). Quantum GIS Geographic Information System. Open Source Geospatial Foundation Project. http://qgis.osgeo.org).

183 African white-backed vultures were fitted with patagial tags, of which 104 birds were captured in KwaZulu-Natal and 79 in the Greater Kruger National Park. Of these birds, 144 (76.7%) were re-sighted 1001 times across the region (Fig. 1) between November 2009 and February 2016, resulting in 326 unique annual re-sightings of these birds.

Tagged AWbVs were re-sighted at 166 different localities in five different countries: South Africa, Botswana, Namibia, Zimbabwe and Zambia (Fig. 1, Table S1). Birds originally tagged in KZN were re-sighted in the KZN region as well as in the Greater Kruger region, with five sightings from further afield in Zimbabwe, Botswana and Namibia. Birds originally tagged in the Greater Kruger region were predominantly re-sighted within this region, with only two birds re-sighted in KZN (representing 0.3% of all resightings of Kruger birds); an additional five birds were seen in Zimbabwe and Zambia to the north (Fig. 1). In contrast 55 birds originally tagged in KZN were resighted in Kruger (24.6% of all resightings of KZN birds).

The best MARK model represented survival varying with age and region (i.e. Kruger or KZN), and recaptures varying with region and time (Table 1), which was well supported (AIC_c_ weight = 0.99419). There were no competing models (Table 1), and the next best model differed by an AIC_c_ >10 (Table 1). The survival estimates (combined across all age classes) for AWbVs in Kruger and KZN were 0.858 ± 0.04823 and 0.683 ± 0.03947, respectively.

**Table 1:** The candidate models used to estimate survival in African white-backed vultures captured in South Africa.

Model	Description	AICc	Delta AICc	Weight	n
Phi(site + age), p(site + t)	Survival varies with region and age, recaptures vary with region and time	619.6521	0	0.99419	18
Phi(site + age), p(t)	Survival varies with region and age, recaptures vary with time	630.4739	10.8218	0.00444	13
Phi(site), p(site + t)	Survival varies with region, recaptures vary with region and time	633.4523	13.8002	0.00100	14
Phi(age), p(site + t)	Survival varies with age, recaptures vary with region and time	637.2545	17.6024	0.00015	15
Phi(site + t), p(site + t)	Survival and recaptures vary with region and time	637.6956	18.0435	0.00012	22
Phi(site + age), p(.)	Survival varies with region and age, recaptures constant	644.6853	25.0332	0.00001	7
Phi(.), p(t)	Survival and recaptures vary with age	645.7124	26.0603	0.00001	8

Estimates of survival (phi) and recapture (p) were modelled with region (site), time (t) and/or age class of the vultures (age). The number of parameters is indicated by ‘n’. The models are arranged from best (top of table) to worst (bottom). Also presented are the AICc values, the difference in AICc value with the top model (Delta AICc) and the model weight.

### Matrix Population Models

We constructed matrix population models using the survival estimates gained from our MARK analysis. Our individual matrix population models for the two populations showed divergent trends in terms of the population growth rate λ. We obtained λ values of 0.65 (declining population) and 1.04 (increasing population) for the KZN and Kruger birds, respectively. The elasticity and sensitivity values obtained show that λ was most sensitive to changes in the survival of adult birds for both the Kruger and KZN populations when considered in isolation, with fecundity the next most important rate (Table 2). A matrix model which allowed for dispersal between the two regions resulted in a positive growth rate of λ = 1.01.

**Table 2:** Sensitivities and elasticities of the vital rates for the two populations from the matrix population models.

Parameter	Kruger			KZN		
	Sensitivity	Elasticity	Reproductive value	Sensitivity	Elasticity	Reproductive value
2^nd^ year survival	0.038	0.030	1.000	0.063	0.083	1.000
3^rd^ year survival	0.038	0.030	1.263	0.063	0.083	0.758
4^th^ year survival	0.035	0.030	1.595	0.107	0.083	0.574
5^th^ year survival	0.035	0.030	1.855	0.107	0.083	0.734
5+ year survival	0.879	0.849	2.158	0.660	0.582	0.938
Fecundity	0.407	0.030	NA	0.710	0.083	NA

The sensitivities represent the partial derivative of the lambda, the population growth rate, with respect to each element, holding all others constant. These values show which vital rates have the biggest impact on lambda. The elasticity values are standardised sensitivities. This is done so that fecundity and survival, which have different units of measurement can be directly compared. Also shown is the reproductive value for each life stage i.e. their expected contribution to the population.

We simulated the effect of poisoning on the population of the Kruger and KZN birds whereby catastrophic poisoning events occurred on average every 5, 10 or 15 years. This was realised by lowering the survival rates of the adults and subadults of Kruger to the same level as we found for their KZN equivalents while still allowing for dispersal between the regions. The simulation was for 100 years (see methods). When this decline occurred every 15 years the overall population had a pseudo-extinction probability of 0.016, when it occurred every 10 years, the extinction probability was 0.202 and when it occurred every 5 years the extinction probability was 0.960. Thus, given these parameter values it is highly probable that the population as a whole will be driven to extinction if Kruger bird survival declines to KZN rates even once every 5 years.

### Agent-based model

In order to explain the differential survival rates of our MARK model, we developed a spatially explicit agent-based model using the NetLogo program. This approach has previously been used to model the spatial ecology of scavengers^18,23^. The model had two variants one where it was centred on a protected area (e.g. Kruger) with non-protected areas outside of this and one that was focussed on non-protected areas (e.g. KZN) with protected areas outside of this. The model parameters are given in Table S3.

**Figure 2:**
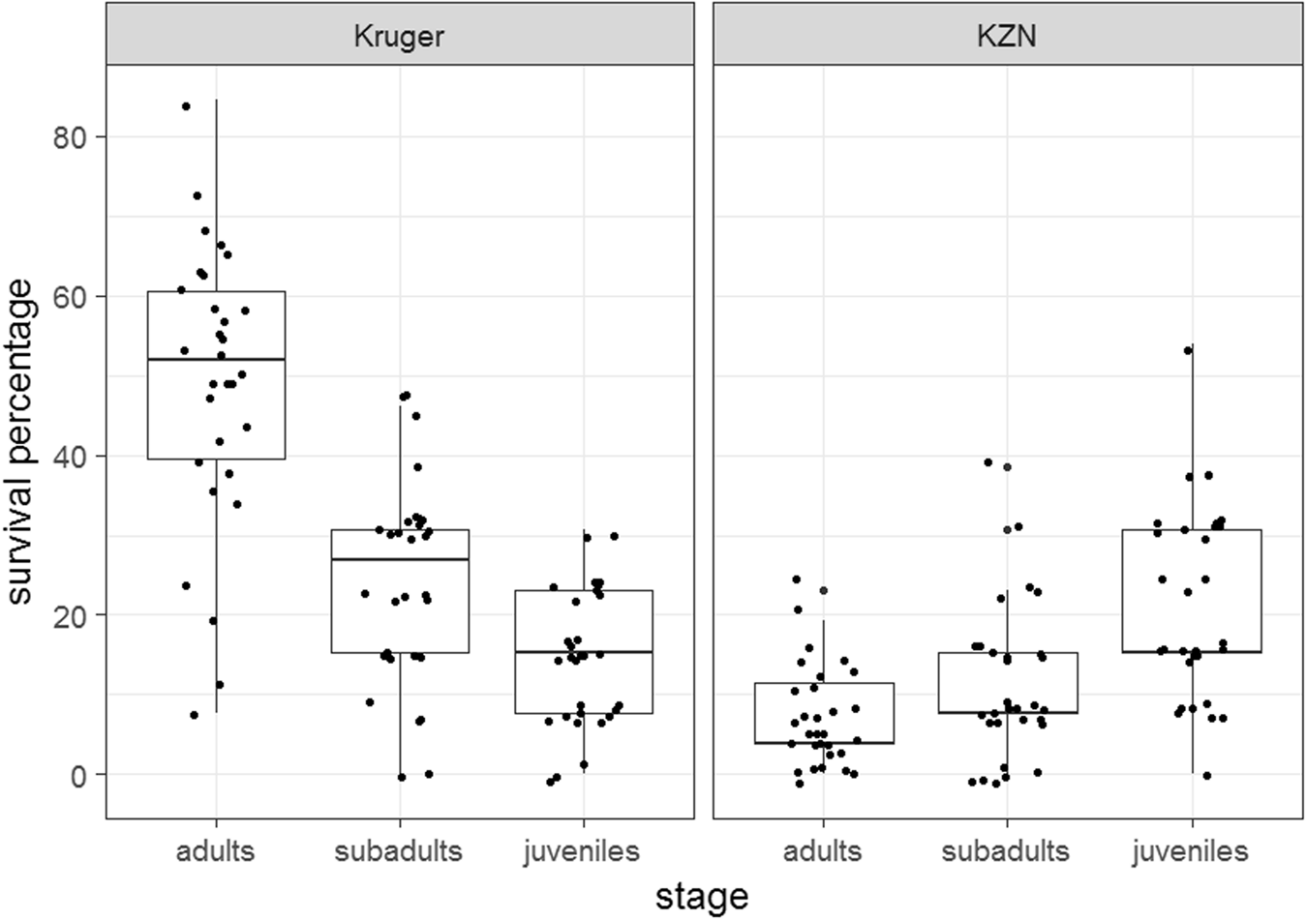
Boxplots showing comparison of vulture life stage survival (as a percentage) in the Kruger focused agent-based model and the KZN focused version. In this case, poison rates were 1 in 1000 for Kruger and 1 in 100 for KZN. This means a carcass has a 1 in 1000 or 1 in 100 chance of being poisoned respectively. Thick horizontal lines represent the median value, the box represents the interquartile range, and the whiskers extend to 1.5 times the interquartile range.

The model, which allowed for spatially variable poisoning rates and realistic movements of the different life stages, showed that Kruger can give a protective effect to the birds therein under scenarios where its poisoning rate is lower than non-protected sites. This can be seen in average modelled survival rates for adult vultures in KZN and Kruger (Table 3). There is also a general pattern of variable survival rates for the three life stages as seen from the summary statistics (Table 3). Juveniles, which were not constrained to a nest, have higher survival rates when the model is focused on KZN and lower survival rates when the model is focused on Kruger (Fig. 2). The unusual empirical result of juvenile survival exceeding that of the adults and subadults in KZN birds is captured by all models. This was the only stage class not tied to the central colony at any point.

**Table 3:** Mean and standard deviation (SD) of Life stage survival (as a percentage) for the different focal locations along with sensitivity analyses of the model parameters of the agent-based model.

Kruger Rate	KZN Rate	Mean adult	Mean juvenile	Mean subadult	SD adult	SD juvenile	SD subadult	Focus	Roosts
1000	100	48.98	14.36	24.36	17.61	8.51	12.47	Kruger	10
1000	100	7.05	21.02	11.28	6.15	12.11	9.42	KZN	10
2000	100	56.54	23.59	28.72	16.23	13.39	14.7	Kruger	10
2000	100	9.49	19.23	16.66	7.87	10.05	9.28	KZN	10
500	100	38.97	16.15	22.05	15.41	10.37	11.2	Kruger	10
500	100	8.33	13.08	10.77	10.93	10.33	10.02	KZN	10
1000	100	45.77	17.43	25.64	17.48	11.24	14.04	Kruger	5
1000	100	11.41	18.97	16.15	11.45	11.73	9.11	KZN	5
1000	100	49.62	22.31	26.41	16.26	13.15	16.98	Kruger	20
1000	100	8.08	17.18	12.05	9.86	10.63	10.24	KZN	20

The “Rate” represents the probability of poisoning where 1000 means a carcass has a 1 in 1000 chance of being poisoned. The “Focus” shows whether the model and hence the vulture colony site was centred on Kruger or KZN. The “Roosts” specifies the number of nesting sites available to the birds apart from the central colony.

We tested for the statistical significance of these patterns. Here we describe the results for the case where the poisoning rate in Kruger was 1 in a 1000 and in KZN was 1 in 100, i.e. where the poisoning rate was 10 times higher in KZN than in Kruger. Firstly, a model of survival ~ area was significant at p < 0.05. (Wilcoxon rank sum test, W = 6119, p < 0.0001).

A Kruskal-Wallis test showed a significant difference between survival rates of the life stages for both Kruger (z = 46.022, df = 2, p < 0.001) and KZN (z = 24.904, df = 2, p < 0.001) simulations.

A subsequent Dunn’s post hoc test showed significant differences at p < 0.05 for all comparisons aside from that of the adult : subadult in KZN. The pattern was generally consistent for the sensitivity analyses we conducted although the significance of the results at p = 0.05 does vary (Table 4).

**Table 4:** Post hoc statistical comparison of survival of different life stages using Dunn’s test for the agent-based model simulations under different parameter values. The p-values are adjusted to account for multiple comparisons.

Kruger Poison Rate	KZN Poison Rate	Comparison	Z score	P.unadjusted	P.adjusted	Focus	N-Roosts
1000	100	adults-juveniles	6.71	<0.05	<0.05	Kruger	10
		adults-subadults	4.24	<0.05	<0.05	Kruger	
		juveniles-subadults	−2.47	<0.05	<0.05	Kruger	
		adults-juveniles	−4.93	<0.05	<0.05	KZN	
		adults-subadults	−1.78	NS	NS	KZN	
		juveniles-subadults	3.15	<0.05	<0.05	KZN	
2000	100	adults-juveniles	6.22	<0.05	<0.05	Kruger	10
		adults-subadults	4.88	<0.05	<0.05	Kruger	
		juveniles-subadults	−1.34	NS	NS	Kruger	
		adults-juveniles	−3.87	<0.05	<0.05	KZN	
		adults-subadults	−2.9	<0.05	<0.05	KZN	
		juveniles-subadults	0.97	NS	NS	KZN	
500	100	adults-juveniles	5.59	<0.05	<0.05	Kruger	10
		adults-subadults	3.93	<0.05	<0.05	Kruger	
		juveniles-subadults	−1.67	NS	NS	Kruger	
		adults-juveniles	−2.3	<0.05	NS	KZN	
		adults-subadults	−1.3	NS	NS	KZN	
		juveniles-subadults	1	NS	NS	KZN	
1000	100	adults-juveniles	5.89	<0.05	<0.05	Kruger	5
		adults-subadults	4.06	<0.05	<0.05	Kruger	
		juveniles-subadults	−1.83	NS	NS	Kruger	
		adults-juveniles	−2.81	<0.05	<0.05	KZN	
		adults-subadults	−2.1	<0.05	NS	KZN	
		juveniles-subadults	0.71	NS	NS	KZN	
1000	100	adults-juveniles	5.37	<0.05	<0.05	Kruger	20
		adults-subadults	4.65	<0.05	<0.05	Kruger	
		juveniles-subadults	−0.72	NS	NS	Kruger	
		adults-juveniles	−3.58	<0.05	<0.05	KZN	
		adults-subadults	−1.74	NS	NS	KZN	
		juveniles-subadults	1.84	NS	NS	KZN	

We compare the different life stages against each other for all five sensitivity analyses. The “Rates” represent the probability of poisoning where 1000 means a carcass has a 1 in 1000 chance of being poisoned. The “Focus” shows whether the model and hence the vulture colony site was centred on Kruger or KZN. The “Roosts” specifies the number of nesting sites available to the birds apart from the central colony.

## Discussion

Our study shows clear demographic differences between AWbVs in two spatially adjacent areas (namely Kruger and KZN), resulting in opposite forecasts for their populations. Based on our estimates, the Kruger birds show typical survival rates for *Gyps* vultures^11,24–26^, and population growth is positive. In contrast, survival rates of KZN birds are far lower than expected for AWbVs^26^, and unsurprisingly, this population is predicted to decline rapidly over the next decade; a result of the low survival rates of subadults and adults. This decline may already be in evidence to some extent, based on long-term monitoring of nests. For example, between 2005 and 2015 the AWbV breeding population at Mkhuze Game Reserve in KZN declined by 58% (unpublished data).

We suggest that a declining population and low survival rates for subadults and adults are a result of differences in the incidence of poisoning, probably the main non-natural mortality factor of vultures^1,16,21^, between Kruger and KZN. By building a spatially explicit simulation model, in which different age classes of vultures range over different geographical areas, as shown for the Cape vulture *Gyps coprotheres*^27,28^, we show that it is possible to achieve the relative differences in age class survival estimated by our tagging study, strongly suggesting that poisoning is responsible. There are two important considerations arising from this observation.

The first important consideration is that the Kruger and KZN birds are separated by a “gap” of just 200 km. This area, which coincides with the country of Swaziland, is not a real gap since there is a large vulture colony breeding here^29^. And in any case, 200 km over land does not represent any real barrier to vulture movements^30^. Indeed, birds could cover this distance in less than a day’s flying^31^, and do so frequently as seen in birds fitted with GPS or satellite transmitters (unpublished data). How then is it possible to get such pronounced differences in survival rates in two adjoining areas? We do not know the answer to this question, but offer the following hypothesis. It is possible that AWbVs show some characteristics of a metapopulation structure as presented by Hanski^32^, and that although individual birds can, and do, move widely^22^, the population is divided into distinct subgroups (akin to metapopulations) to which breeding birds will consistently return. Testing this hypothesis is important because, if shown to be correct, there would be major implications for the conservation of vultures, a group of birds that is disproportionately at risk of extinction^15,33^, yet plays a key ecological role through nutrient recycling and carcass removal^34^. For example, one conservation management approach to deal with the Asian vulture crisis is the implementation of “vulture safe zones” - areas surrounding critical breeding colonies from which non-steroid anti-inflammatory drugs are completely removed and drug-free food is provisioned at safe vulture restaurant sites^35^. The efficacy of such vulture safe zones remains to be tested, and in fact the concept has only recently been suggested in an African context^36^. However, this study provides the first evidence that such an approach might be effective in an African context whereby important breeding colonies and the areas around them could be targeted with direct conservation action, such as anti-poisoning strategies^21^ to reduce the illegal use of poisons and secure their long-term survival. As a first step, the size of these areas could be based on the size of area relevant to breeding adults, which would require protecting a circle with a radius equal to their foraging range (~55 km)^31^.

The second important consideration is the juvenile survival rate in the KZN region, which greatly surpasses that of adults or subadults. We suspect this is due to adult birds being tied to a nest as they begin to breed. Indeed, previous work, which involved tracking actively breeding AWbVs, showed that the maximum distance the birds travelled away from their starting point was approximately 55 km^31^. This emphasises further the need for direct conservation actions around breeding sites. Younger birds, however, are free to range widely and suffer no such constraint^37^. As a result, young birds from KZN are traveling further afield to areas where their survival might be enhanced (or at least not reduced) by lower levels of poisoning. Furthermore, young and inexperienced birds may be drawn to vulture restaurants whereas older (and more experienced) adult birds are less inclined to forage at restaurants and may therefore be more readily exposed to poisoned carcasses^38^. Thus, whilst young KZN birds may be spared the high rates of poisoning affecting the adult population, the provision of vulture restaurants alone might be largely ineffective at protecting breeding adults. Why the subadults in KZN suffer a similar fate to that of adults is not known and deserves further investigation but may relate to prospecting behaviour of the subadults whilst looking for suitable breeding areas.

A caveat of this study is that there are other serious threats facing the AWbV, any one of which may have influenced the survival rates estimated for Kruger and KZN birds. These threats include: trade in traditional medicine; killing for human consumption; and collision/electrocution with electrical power infrastructure^15^. The trading of vulture parts in traditional medicine is rife in southern Africa, with several large markets serving as major outlets^19,20,39^. However, most of these birds appear to be acquired by poisoning^15^ and therefore would fall under our approach in this study. With regard to the second serious threat, vultures are primarily eaten in West and Central Africa^15,40^ but this has not been documented in southern Africa. Finally, there is no reason to suggest that vultures in Kruger and KZN face inherently different risks from powerline infrastructure. A second caveat is that we do not have accurate or reliable estimates of the incidence of poisoning at either of our two study regions; hence our findings need to be interpreted with caution. On the one hand, we urge researchers to test the outcome of our model suggesting higher incidence of poisoning in KZN compared with Kruger, as well as differential resource utilization by different aged birds. On the other hand, we emphasize that our study has demonstrated the potential dramatic effecting of poisoning on adult mortality which itself has devastating impacts on future population trends.

In terms of the modelling approach, future work should allow us to build in more realistic dispersal rates for the different age classes and include mortality measures from other sources. As it stands, our agent-based model can only show relative differences in survival. More studies involving high resolution tracking data of vulture populations will inevitably allow for better parameterisation.

In conclusion, we present the first evidence that vulture demography may vary considerably over relatively small spatial scales. These findings may have significant consequences for vulture conservation, in particular the potential setting up of “vulture safe zones” and providing targeted, spatially explicit conservation action to address the risks from poisoning.

## Methods

### Ethics statement

All the work was conducted in accordance with relevant national and international guidelines, and conforms to all legal requirements. All experimental protocols were approved and endorsed by the Endangered Wildlife Trust Ethics Committee. Furthermore, vulture captures were carried out in compliance with the South African National Parks and EKZN Wildlife Scientific Services, South Africa. Wing-tagging of vultures was conducted within the guidelines adopted by the Birds of Prey Working Group of the Endangered Wildlife Trust.

### Study sites and study design

African white-backed vultures were captured at two separate localities in South Africa: 1) the Greater Kruger National Park (combining the Kruger National Park with adjoining conservation areas to the west) situated in the Mpumalanga and Limpopo Provinces, and referred to as the “Kruger” site; and 2) in the KwaZulu-Natal Province, referred to as the “KZN” site. Vultures were captured widely across the Greater Kruger National Park area at 30 different trapping sites (Figure S1), although almost half of all captures were at two localities: Moholoholo Rehabilitation Centre (24° 31′S; 30° 54′N), and the Pafuri airstrip (22° 25′S; 31° 13′N). Vultures were captured at three protected areas in KwaZulu-Natal: Hluhluwe-iMfolozi Park; uMkhuze Section iSimangaliso Park; and Phongolo Nature Reserve (including the adjoining Zimanga Game Reserve and Zululand Rhino Reserve); as well as a fourth site, the Kempenfeldt Vulture Restaurant (Figure S1). Kruger birds were captured between October 2011 and February 2016, whereas KZN birds were captured between November 2009 and January 2016. The sample sizes of different age-classes captured in each year from each region is presented in Table S1.

Although poisoning incidents have occurred at both these areas (Kruger and KZN)^41–43^, the actual intensity of poisoning is not known. The Endangered Wildlife Trust (Johannesburg, South Africa) keeps a record of all poisoning incidents that have involved vultures; some 871 vultures were reported to have died from 41 poisoning incidents in the Kruger and KZN areas between 2009 and 2015, inclusive (EWT, unpublished data). This confirms that high levels of poisoning do occur in this region, however, due to the haphazard way in which such events are reported^7^, it is not possible to use these data to estimate the incidence of poisoning at these two sites. Instead, we take an alternative approach to address this question by first calculating the survival rate of birds in these two regions, and then developing a spatially explicit simulation model of poisoning events. The model, which reflects the known biology of the AWbVs, is then correlated with the pattern in survival rates that we estimated (see below for details).

### Data collection

Vultures were captured either as chicks on the nest, or as adults in a specially-designed noose trap. Nests were accessed using a cherry-picker vehicle to access nests quickly and efficiently. Nestlings were removed from the nest and processed on the ground before being returned to the nest. Free-flying birds of varying ages were captured at baited carcasses using foot-nooses, a commonly used method to capture individuals or small numbers of birds at a trapping site^44,45^.

All captured birds were tagged, aged and measured before being released at point of capture. Ageing was based on plumage characteristics^46^ and birds were assigned to one of three age classes: juvenile (first and second-year old birds), subadult (third-year to fifth-year old birds), and adults (six years old or older). Sexing of AWbVs based on external features has not been reliably described. Thus, birds were not assigned a sex in the field, and sex was dropped as a feature in subsequent analyses. Each bird was fitted with a metal ring issued by AFRING (Animal Demography Unit, University of Cape Town) and a patagial tag. Patagial tags were fitted according to the standard protocol adopted for this practice in southern Africa^47^. It involved the use of a double set of standard cattle tags engraved with a unique number which was fitted to the patagium on each wing of each bird using a tag applicator. This method was extensively assessed prior to this study and found not to be detrimental to the birds’ health or inhibiting their ability to forage^47^. All tagged AWbVs were released unharmed and immediately after processing each individual which took, on average, 6 min per bird.

A dedicated re-sightings programme was established by publicising the project using television and radio broadcasts, articles to local newspapers and magazines, and posters in various rest camps in the Greater Kruger National Park as well as parks managed by Ezemvelo KZN Wildlife. Re-sightings were reported by a wide range of people by phone, e-mail or sms. A significant proportion of re-sightings was submitted by the staff at two vulture feeding sites: (1) Moholoholo near the town of Hoedspruit in the Greater Kruger National Park region; and (2) Kempenfeldt near the town of Dundee in the KZN region. Re-sightings were also reported by managers of other vulture restaurants, game ranchers, farmers and tourists.

### Data analysis

Survival and recapture were computed, using the standard Cormack-Jolly-Seber model, in the program MARK^48^ using capture-resightings of AWbVs. This model is appropriate since just two tagged vultures were recovered dead and which were excluded from the analysis. We used a one-year interval with all birds scoring either “1” (seen) or “0” (not seen) in each year. Our observation period extended over the entire year which violates the assumptions of capture-recapture analysis^49^. However, this may not necessarily bias survival estimates and may in fact be appropriate for species for which large sample sizes are not easily obtainable, by reducing precision of the estimates^50,51^. A variety of models that included time dependence, region (i.e. Kruger and KZN), and age were developed; where “region” was treated as a group. Models were ranked using Akaike’s Information Criterion (AIC). The model with the lowest AICc was deemed the best model; where ∆AICc (the difference in AICc between models) for any two (or more) models was <2.0, they were both deemed to be equally good. The location of re-sightings was plotted using QGIS. All mean values are quoted with ±SE.

The loss of tags from vultures and other large birds have been reported from other recent studies using these patagial tags^12,26^, particularly after five years. Since there is no reason to suspect that the rate of tag loss would differ between the Kruger region and the KZN region, any errors in estimation of survival rates should equally affect both regions.

### Population dynamics models - Individual populations

We used a population dynamics model to determine the population growth rate (designated as λ) of the birds in the two regions^52^. We took the survival estimates from the MARK analysis to initially create two, stage-structured, matrix population models, one using the estimates for the Kruger birds and the other using estimates for the KZN birds. The age classes in the model are chicks (0–1 yr old = s0), juveniles (1–3 yr old = s1), subadults (3–5 yr old = s2) and adults (5+ = s3). Thus, we had two 5 × 5 matrices of the form (where subscript i gives the region Kruger or KZN):

$$(\begin{array}{ccccc}0 & 0 & 0 & 0 & {f}_{i}\\ s{1}_{i} & 0 & 0 & 0 & 0\\ 0 & s{1}_{i} & 0 & 0 & 0\\ 0 & 0 & s{2}_{i} & 0 & 0\\ 0 & 0 & 0 & s{2}_{i} & s{3}_{i}\end{array})$$

These matrix models operate by multiplying a vector of population sizes by a matrix of stage-specific survival and fecundity, termed the vital rates. This produces a new vector of population sizes at time t + 1. This process is iterated to determine the population growth rate λ, which is the dominant eigenvalue of the matrix. We also calculated the sensitivity and elasticity (standardised sensitivity) of the vital rates (see Table S2). This was done by taking the partial derivative of λ with respect to each vital rate element. They were standardised in order to make direct comparisons between survival rates which are bounded between 0 and 1 and fecundity measures which are not bounded in this way. The resulting values were used to determine which vital rate had the greatest impact on λ^52^.

Our models assume a pre-breeding census^52^ (the youngest age class are 1 year old birds) and we took literature estimates to set the other parameters namely: fecundity; first year survival; and population size. Here, following Gauthier & Lebreton^53^, we define fecundity (f_i_) as breeding propensity * (clutch size/2) * hatching success * fledging success * first year survival. Clutch size was divided by 2 because these models typically focus on females by convention. African white-backed vultures reach breeding age at 5 years.

There are an estimated 1200 and 425 female birds in Kruger and KZN respectively^21,43,54^. We divided these up among our age classes using the estimated distribution of these in a vulture population i.e. 9% of birds are under 2 years old, 24% are between 3 and 5 years old and adults aged over 5 years old represent 67% of the population^21^.

### Population dynamics models–Metapopulation

We then combined these matrices into a metapopulation structure to allow us to incorporate dispersal between the populations^55^ resulting in a 10 × 10 matrix. Here, the Kruger population is represented in the upper left, the KZN population in the lower right and the dispersal rates in the diagonals. The rates designated ‘gb’ and ‘bg’ represent the dispersal rates from Kruger to KZN and KZN to Kruger respectively:

$$(\begin{array}{cccccccccc}0 & 0 & 0 & 0 & {f}_{Kr}(1-gb0) & 0 & 0 & 0 & 0 & {f}_{Kr}bg0\\ s{1}_{Kr}(1-gb) & 0 & 0 & 0 & 0 & s{1}_{zr}bg & 0 & 0 & 0 & 0\\ 0 & s{1}_{Kr}(1-gb) & 0 & 0 & 0 & 0 & s{1}_{Kz}bg & 0 & 0 & 0\\ 0 & 0 & s{2}_{Kr}(1-gb) & 0 & 0 & 0 & 0 & s{2}_{Kz}bg & 0 & 0\\ 0 & 0 & 0 & s{2}_{Kr}(1-gb) & s{3}_{Kr}(1-gb) & 0 & 0 & 0 & s{2}_{Kz}bg & s{3}_{Kr}bg\\ 0 & 0 & 0 & 0 & {f}_{Kr}gb0 & 0 & 0 & 0 & 0 & {f}_{Kz}(1-bg0)\\ s{1}_{Kr}gb & 0 & 0 & 0 & 0 & s{1}_{Kr}(1-bg) & 0 & 0 & 0 & 0\\ 0 & s{1}_{Kr}gb & 0 & 0 & 0 & 0 & s{1}_{Kz}(1-bg) & 0 & 0 & 0\\ 0 & 0 & s{2}_{Kr}gb & 0 & 0 & 0 & 0 & s{2}_{Kz}(1-bg) & 0 & 0\\ 0 & 0 & 0 & s{2}_{Kr}gb & s{3}_{Kr}gb & 0 & 0 & 0 & s{2}_{Kz}(1-bg) & s{3}_{Kz}(1-bg)\end{array})$$

We assumed dispersal was equal in either direction, that 5% of first year birds migrated and 2% of the remaining stages did so^21^. We again calculated the population growth rate λ.

### Population dynamics models-Pseudo-extinction probability

We then calculated the pseudo-extinction probability for these two linked populations, this is the point at which the population can be considered as critically endangered or essentially extinct^56^. We looked at how the population would respond if the Kruger sub-populaton vital rates were changed to match those of the KZN sub-population. In particular, we lowered the subadult (s2_Kr_) and adult (s3_Kr_) survival rates to match those of the equivalent KZN rates, all other parameters being kept constant. Following Soldatini *et al*.^57^, we implemented these reductions in survival using a Bernouli distribution under three different scenarios. We considered that the reductions could occur on average: (1) once every 5 years; (2) once every 10 years; and (3) once every 15 years. Note that, outside of these years, the surivial rates return to their original higher values. We set the threshold for extinction at 10 females and ran the simulation for 100 years. This allowed us to determine the extinction probability as the number of times the population fell below 10. We conducted this analysis using the “popbio” package in R version 3.3.1. The R code for the population dynamics calculations can be accessed at the following link: https://github.com/kanead/white-backed-vulture-population-dynamics/blob/master/Matrix%20Models/Vulture%20Metapopulation%20pseudo-extinction%20probability.R.

### Agent-based model–The model

The full model can be accessed at the following link: https://raw.githubusercontent.com/kanead/white-backed-vulture-population-dynamics/master/vulture%20poison%20model.nlogo.

### Agent-based model-The Environment

The environment in the simulation is a patchwork of squares which is inherent to NetLogo. For our model, each patch represents a 1× 1 km square. We set out a circle within the simulation space which is equivalent to 20,000 km^2^. The area outside of this circle is equal in size (20,000km^2^ is the approximate size of Kruger). There are two habitat types, the protected area, which represents Kruger, and a non-protected area which represents KZN. We can flip the focus of the model such that the circle represents either Kruger or KZN. A small inner circle represents the roost, with an area of 200 km^2^ and radius 8 km. A medium-sized middle circle has a 50 km radius and represents the foraging range of white-backed vultures^31^. There are specific patches, randomly distributed around the model at the setup stage, which act as roosts for juveniles, subadults and later on the adults. We vary their number as a form of sensitivity analysis.

### Agent-based model-The Vultures

There were three classes of vulture in the model which represent the ages that we were interested in: adults, subadults, and juveniles. The adults get distributed randomly within the inner circle at the setup stage. Their number is set to 26 birds because of the density reported by^58^ (13 birds per 100 km^2^). The subadults and juveniles are randomly distributed around the whole space at the setup stage. They each have a population size of 13 birds as it will take a pair of adults to produce a member of the younger age class.

Once the model is initiated the birds start moving at their set speed of 24 km/hr, (this is written in the model in km per second = 0.00667). They randomly turn left or right by 15° every 10 min. Initially they head off at a random direction but this can change once they encounter food (see below). The adults are initially restricted to move within the medium-sized circle which is their foraging radius but the other two classes do not have any such restrictions.

In the absence of food, the birds move around for 9 hrs and return to their roost after this point. They double their speed to 48 km/hr so they get home in time. The model goes on a bit longer than 9 hrs to allow for them to return home. The day length is thus 11 hrs (39600 s). Adults return to the central colony where they started. Juveniles return to the nearest roost patch. Subadults have a 50:50 chance of moving to the central colony (this represents prospecting for nest sites) or the nearest roost patch. Adults are free from the restriction of the colony after 8 months (day 240), after which point they move towards the roosts like juveniles^46^.

The birds have a set vision of 6 km, which means that they can detect a carcass six patches away^59^. Once they do, they move towards it. If a bird lands on a carcass it stays there until the 9 hrs elapse, then it goes straight home. While a bird is on a carcass it creates a local enhancement effect such that other birds can now see the carcass from 7 km away rather than 6 km *a la* Jackson *et al*.^60^. A large carcass (>1000 kg) is always visible from 7 km away. If a bird lands on a poisoned carcass it dies immediately.

The birds have a memory of the location of the carcasses they feed on. So, aside from day 1, they move towards the patch that had the carrion they fed upon the previous day. However, if they pass a different carcass on the way, they will feed on that one instead.

### Agent-based model–Carcasses

Carcasses are distributed randomly throughout the environment. But the carcass density is different for the two areas. For the protected area it is 0.15 kg of carcass per km^2 ^^58^ * area of park (20,000 km^2^) = 3,000 kg carrion in the protected area. The carrion is packaged up into carcasses of a size set by a habitat-specific distribution. In Kruger carrion is distributed according to a Gamma distribution which allows for the occasional large carcass. The Gamma parameters are set at alpha = 1.2 and beta = 0.004. The probability of a carcass being large (>1000 kg) is a little under 3% (Probability (X >x) = 0.02745).

For the non-protected area, the density is higher at 0.3 kg per km^2^. This is distributed according to a normal distribution (mu = 500 kg, sd = 100 kg) because there are not the extremes of body size outside of the protected area.

Carcasses less than 1000 kg decay after a day but carcasses greater than 1000 kg take two days to decay. This is included because the birds have memory and will move back towards the food. A large carcass that happens to be poisoned will do more damage this way. Large carcasses are also visible from further away. Each day a new batch of carcasses are randomly distributed around the map up to the maximum density set in the model for the two areas of Kruger (3000 kg) and KZN (6000 kg) aside from the large carcasses which persist for 2 days. Overall, the environment has just over 9 tons of carrion.

### Agent-based model–Poisoning

The rates of poisoning are set to differ between the two areas, protected and non-protected. A value of 100 means a 1 in 100 chance of a carcass being poisoned. These are the focus of our sensitivity analyses because data on poisoning rates are not available; hence we were interested in relative differences.

### Agent-based model–Simulation

The models lasted for 365 simulation days to capture annual variability in vulture behaviour especially with respect to the adults. For given poison rates we ran two model variants, one with Kruger as the focus and one with KZN as the focus. Each of these variants was repeated 30 times. Thus, we had 30 runs each lasting a year in the simulation for a Kruger focused model and the same for a KZN focused model. Refer to Tables 3 and 4 for all model runs. We analysed the output of the agent-based model as survival ~ age class for each region i.e. KZN and Kruger. Visual inspection of the residuals of a model after an ANOVA (survival ~ age class) showed a strong deviation from normality, thus we used a non-parametric alternative (a Kruskal-Wallis test).

## Electronic supplementary material

Supporting Information

## References

[1] Ogada DL, Keesing F, Virani MZ (2012). Dropping dead: causes and consequences of vulture population declines worldwide. Ann. N. Y. Acad. Sci..

[2] Wenny DG (2011). The need to quantify ecosystem services provided by birds. Auk.

[3] Markandya A (2008). Counting the cost of vulture decline-An appraisal of the human health and other benefits of vultures in India. Ecol. Econ..

[4] Phipps, W. L., Wolter, K., Michael, M. D., MacTavish, L. M. & Yarnell, R. W. Do power lines and protected areas present a catch-22 situation for Cape Vultures (Gyps coprotheres)? *PLoS One* **8** (2013). 10.1371/journal.pone.0076794 PMC3793913 24137496

[5] Phipps WL (2017). Due South: A first assessment of the potential impacts of climate change on Cape vulture occurrence. Biol. Conserv..

[6] Cortés-Avizanda A, Colomer MÀ, Margalida A, Ceballos O, Donázar JA (2015). Modeling the consequences of the demise and potential recovery of a keystone-species: wild rabbits and avian scavengers in Mediterranean landscapes. Sci. Rep..

[7] Ogada DL (2014). The power of poison: pesticide poisoning of Africa’s wildlife. Ann. N. Y. Acad. Sci..

[8] Green RE (2004). Diclofenac poisoning as a cause of vulture population declines across the Indian subcontinent. J. Appl. Ecol..

[9] Oaks JL (2004). Diclofenac residues as the cause of vulture population decline in Pakistan Diclofenac residues as the cause of vulture population decline in Pakistan. Nature.

[10] Margalida A, Colomer MÀ, Oro D, Arlettaz R, Donázar JA (2015). Assessing the impact of removal scenarios on population viability of a threatened, long-lived avian scavenger. Sci. Rep..

[11] Monadjem A, Wolter K, Neser W, Kane A (2014). Effect of rehabilitation on survival rates of endangered Cape vultures. Anim. Conserv..

[12] Monadjem, A., Kane, A., Botha, A., Dalton, D. & Kotze, A. Survival and population dynamics of the marabou stork in an isolated population, Swaziland. *PLoS One* **7** (2012). 10.1371/journal.pone.0046434 PMC3460887 23029517

[13] Margalida A (2014). One health approach to use of veterinary pharmaceuticals. Science (80)..

[14] Margalida A (2012). Baits, budget cuts: a deadly mix. Science (80)..

[15] Ogada D (2016). Another continental vulture crisis: Africa’s vultures collapsing toward extinction. Conservation Letters.

[16] Ogada D, Botha A, Shaw P (2015). Ivory poachers and poison: drivers ofAfrica’s declining vulture populations. Oryx.

[17] Kendall CJ, Virani MZ (2012). Assessing mortality of African vultures using wing tags and GSM-GPS transmitters. J. Raptor Res..

[18] Kane, A., Jackson, A. L., Ogada, D. L., Monadjem, A. & McNally, L. Vultures acquire information on carcass location from scavenging eagles. *Proc. R. Soc. B* **281** (2014). 10.1098/rspb.2014.1072 PMC4173674 25209935

[19] Williams, V. L., Cunningham, A. B., Kemp, A. C. & Bruyns, R. K. Risks to birds traded for African traditional medicine: a quantitative assessment. *PLoS One* **9** (2014). 10.1371/journal.pone.0105397 PMC4146541 25162700

[20] McKean S (2013). The impact of traditional use on vultures in South Africa. Vulture News.

[21] Murn, C. & Botha, A. A clear and present danger: impacts of poisoning on a vulture population and the effect of poison response activities. *Oryx* 1–7 10.1017/S0030605316001137 (2017).

[22] Phipps, W. L., Willis, S. G., Wolter, K. & Naidoo, V. Foraging Ranges of immature African white-backed vultures (Gyps africanus) and their use of protected areas in southern Africa. *PLoS One* **8** (2013). 10.1371/journal.pone.0052813 PMC3559650 23382824

[23] Kane A, Healy K, Ruxton GD, Jackson AL (2016). Body size as a driver of scavenging in theropod dinosaurs. Am. Nat..

[24] Schaub M, Zink R, Beissmann H, Sarrazin F, Arlettaz R (2009). When to end releases in reintroduction programmes: demographic rates and population viability analysis of bearded vultures in the Alps. J. Appl. Ecol..

[25] Gouar PL (2008). Roles of survival and dispersal in reintroduction success of griffon vulture (Gyps fulvus). Ecol. Appl..

[26] Monadjem A, Botha A, Murn C (2012). Survival of the African white-backed vulture Gyps africanus in north-eastern South Africa. Afr. J. Ecol..

[27] Bamford AJ, Diekmann M, Monadjem A, Mendelsohn J (2007). Ranging behaviour of Cape Vultures Gyps coprotheres from an endangered population in Namibia. Bird Conserv. Int..

[28] Monsarrat S (2013). How predictability of feeding patches affects home range and foraging habitat selection in avian social scavengers?. PLoS One.

[29] Monadjem A, Garcelon DK (2005). Nesting distribution of vultures in relation to land use in Swaziland. Biodivers. Conserv..

[30] Lieury N, Gallardo M, Ponchon C, Besnard A, Millon A (2015). Relative contribution of local demography and immigration in the recovery of a geographically-isolated population of the endangered Egyptian vulture. Biol. Conserv..

[31] Spiegel O, Getz WM, Nathan R (2013). Factors influencing foraging search efficiency: why do scarce lappet-faced vultures outperform ubiquitous white-backed vultures?. Am. Nat..

[32] Hanski I (2001). Spatially realistic theory of metapopulation ecology. Naturwissenschaften.

[33] Ogada DL, Torchin ME, Kinnaird MF, Ezenwa VO (2012). Effects of vulture declines on facultative scavengers and potential implications for mammalian disease transmission. Conserv. Biol..

[34] Sekercioglu CH (2006). Increasing awareness of avian ecological function. Trends Ecol. Evol..

[35] Chaudhary A (2012). Population trends of Critically Endangered Gyps vultures in the lowlands of Nepal. Bird Conserv. Int..

[36] Botha, A. J. *et al*. *Multi-species Action Plan to Conserve African-Eurasian Vultures* (2017).

[37] Kane A (2016). Home range and habitat selection of Cape Vultures Gyps coprotheres in relation to supplementary feeding. Bird Study.

[38] Oro D, Margalida A, Carrete M, Heredia R (2008). Testing the goodness of supplementary feeding to enhance population viability in an endangered vulture. PLoS One.

[39] Whiting MJ, Williams VL, Hibbitts TJ (2011). Animals traded for traditional medicine at the Faraday market in South Africa: species diversity and conservation implications. J. Zool. London.

[40] Ogada DL, Buij R (2011). Large declines of the Hooded Vulture Necrosyrtes monachus across its African range. Ostrich.

[41] van Jaarsveld J (1986). Poisoned white-backed vultures in the Kruger National Park. Vulture News.

[42] van Jaarsveld J (1987). Increasing numbers of vultures poisoned in the Kruger National Park. Vulture News.

[43] Rushworth, I. KZN Vulture conservation strategy (2007).

[44] Watson RT, Watson CRB (1985). A trap to capture bateleur eagles and other scavenging birds. South African J. Wildl. Res..

[45] Bloom, P. H., Clarke, W. S. & Kidd, J. W. In Raptor research and management techniques (eds Bird, D. M. & Bildstein, K. L.) (Raptor Research Foundation/Hancock House Publishers, 2017).

[46] Mundy, P. J., Butchart, D., Ledger, J. A. & Piper, S. E. *The Vultures of Africa*. (Acorn books and Russel Friedman books, 1992).

[47] Botha A (2007). A review of colour-marking techniques used on vultures in southernAfrica. Vulture News.

[48] White GC, Burnham KP (1999). Program MARK: survival estimation from populations of marked animals. Bird Study.

[49] Lebreton AJ (1992). Modeling survival and testing biological hypotheses using marked animals: a unified approach with case studies. Ecol. Monogr..

[50] Brien SO, Robert B, Tiandry H (2005). Consequences of violating the recapture duration assumption of mark – recapture models: a test using simulated and empirical data from an endangered tortoise population. J. Appl. Ecol..

[51] Mihoub J-B (2013). Comparing the effects of release methods on survival of the Eurasian black vulture Aegypius monachus reintroduced in France. Oryx.

[52] Caswell, H. *Matrix population models*. (John Wiley & Sons, Ltd, 2001).

[53] Gauthier G, Lebreton J-D (2004). Population models for Greater Snow Geese: a comparison of different approaches to assess potential impacts of harvest. Anim. Biodivers. Conserv..

[54] Murn C, Combrink L, Ronaldson GS, Thompson C, Botha A (2013). Population estimates of three vulture species in Kruger National Park, South Africa. Ostrich.

[55] Hunter CM, Caswell H (2005). The use of the vec-permutation matrix in spatial matrix population models. Ecol. Model..

[56] Brigham, C. A. & Schwartz, M. W. *Population viability in plants: conservation, management, and modeling of rare plants*. (Springer-Verlag, 2003).

[57] Soldatini C, Albores-barajas Y, Massa B, Gimenez O (2016). Forecasting ocean warming impacts on seabird demography: a case study on the European storm petrel. Mar. Ecol. Prog. Ser..

[58] Murn C, Anderson M (2008). Activity patterns of African White-backed Vultures Gyps africanus in relation to different land-use practices and food availability. Ostrich.

[59] Kane, A. & Kendall, C. J. Understanding how mammalian scavengers use information from avian scavengers: cue from above. *J. Anim. Ecol*. 10.1111/1365-2656.12663 (2017). 10.1111/1365-2656.12663 28295318

[60] Jackson AL, Ruxton GD, Houston DC (2008). The effect of social facilitation on foraging success in vultures: a modelling study. Biol. Lett..

