# Supplementary material for: Spatially explicit poisoning risk affects survival rates of an obligate scavenger
Source: Sci Rep. 2018 Mar 12;8:4364. doi: 10.1038/s41598-018-22632-y (PMC5847520; doi:10.1038/s41598-018-22632-y)
Supplement: Supplementary file 1 — Supporting Information [file 41598_2018_22632_MOESM1_ESM.pdf]

Supporting Information

Spatially explicit poisoning risk affects survival rates of an obligate scavenger

A. Monadjem, A. Kane, A. Botha, C. Kelly & C. Murn

Figure S1. Map of the localities at which African white-backed vultures were captured in South Africa. The localities within the Greater Kruger National Park region are shown in black circles, and those within KZN in grey circles. This map was created in Quantum GIS (Quantum GIS Development Team (2016). Quantum GIS Geographic Information System. Open Source Geospatial Foundation Project. http://qgis.osgeo.org).

Figure S1

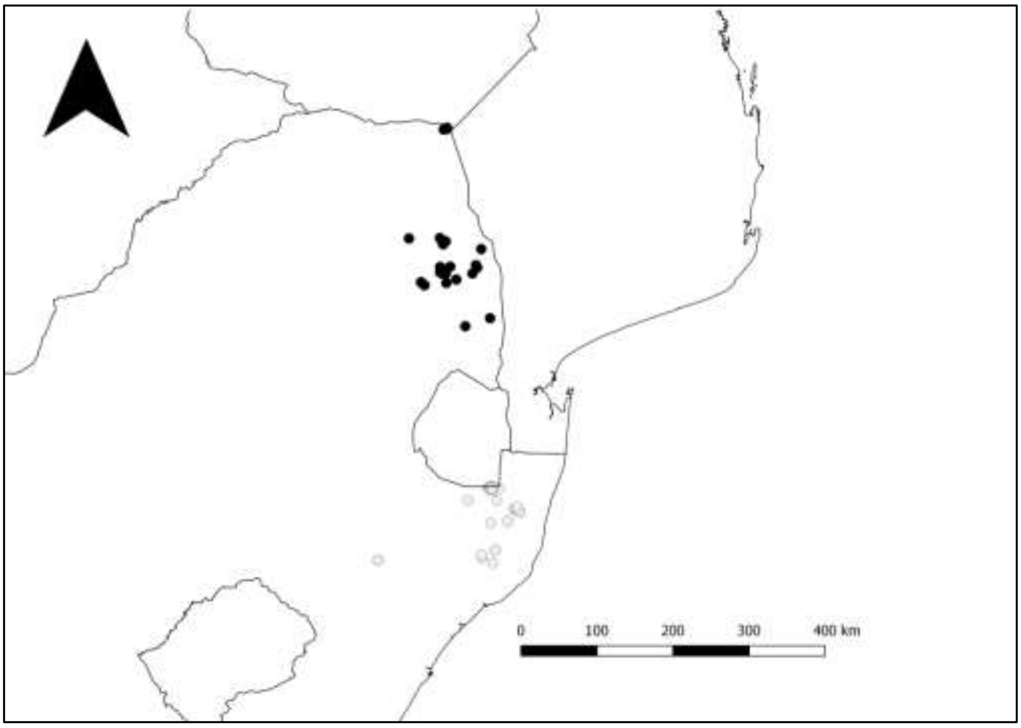

Table S1. The number of juvenile, subadult and adult African white-backed vultures captured and tagged each year in the greater Kruger National Park (Kruger) and KwaZulu-Natal (KZN) regions of South Africa. Also shown are the numbers of birds resighted in each age-class.

Region	Age class	2009	2010	2011	2012	2013	2014	2015	2016	Number resighted
Kruger	Juvenile			4	2	21	3			22
	Subadult					7	18	5	2	26
	Adult					8	7	1	1	12
KZN	Juvenile	8	24	9	7	15	5	6		61
	Subadult	8	4			6	2	3	1	19
	Adult					2		2	2	3

Table S2. Vital rates and values used to construct them for the matrix population models.

**Parameter**	**Kruger birds**	**KZN birds**	**Reference**
Breeding propensity	0.85	0.85	[14]
Clutch size	1	1	[30]
Hatching success	0.76	0.76	[42]
Fledging success	0.6	0.6	[43,44]
First year survival (S_1)	0.42	0.42	[45]
Juvenile survival (S_2)	0.8193305	0.8601882	This study
Sub-adult survival (S_3)	0.8885506	0.5134050	This study
Adult survival (S_4)	1.0	0.5672604	This study

Table S3. Parameter values and associated references for the agent-based model. Comma separated parameter values reflect the sensitivity analyses. See methods and full model in supplementary information for details.

Parameter	Value	Reference
N-adults	26	Murn & Anderson (2008)
N-subadults	13	Murn & Anderson (2008)
N-juveniles	13	Murn & Anderson (2008)
N-roosts	5, 10, 20	This study
Vision	6 km	Kane & Kendall (2017)
Local enhancement effect	Vision + 1 = 7 km	Kane & Kendall (2017)
Speed	24 km/hr	Spiegel et al. (2013)
Foraging time	9 hrs	Spiegel et al. (2013)
Simulation space	40,000 km^2	This study
Adult foraging radius	50 km	Spiegel et al. (2013)
Kruger carrion density	0.15 kg/km^2	Murn & Anderson (2008)<br>Murn pers. comm
KZN carrion density	0.3 kg/km^2	Murn & Anderson (2008)<br>Murn pers. comm
Kruger carrion mass distribution	Gamma(alpha = 1.2 and beta = 0.004)	This study
KZN carrion mass distribution	Normal(mean = 500, SD=100)	This study
Poison rate inside the park	1 in 500; 1 in 1000; 1 in 2000	This study
Poison rate outside the park	1 in 100	This study
Model run	365 days	This study
